# Subchondral bone density changes of the talus in dogs with tarsocrural osteochondrosis

**DOI:** 10.1186/s12917-025-04683-2

**Published:** 2025-04-07

**Authors:** Yasamin Vali, Walter Dingemanse, Magdalena Müller-Gerbl, Eberhard Ludewig, Henri van Bree, Ingrid Gielen

**Affiliations:** 1 Diagnostic Imaging, Department of Companion Animals and Horses, University of Veterinary Medicine Vienna (Vetmeduni), Veterinärplatz 1, Vienna, 1210 Austria; 2 Bristol, UK; 3 Department of Biomedicine, Institute of Anatomy, Basel University, Basel, Switzerland; 4 VetMedImage, Erondegem, Belgium; 5 Department of Morphology, Imaging, Orthopedics, Rehabilitation and Nutrition, Ghent University, Merelbeke, Belgium

**Keywords:** Osteochondritis dissecans, Subchondral bone density, Computed tomography, Canine tarsocrural joint, Computed Tomography osteoabsorptiometry

## Abstract

**Background:**

Osteochondritis dissecans (OCD) and osteochondrosis (OC) are multifactorial developmental joint diseases that can occur in various anatomical locations, including the tarsus of immature, rapidly growing large breed dogs. The pathogenesis of canine OCD and OC involves a disruption in endochondral ossification, resulting in a failure of matrix calcification and vascular invasion. This study aimed to investigate the subchondral bone density changes in Labrador Retrievers with tarsocrural OCD/ OC.

**Results:**

A total of 8 dogs with unilateral tarsocrural OCD/ OC were included in the study and density was evaluated with Computed Tomography osteoabsorptiometry (CTOAM ). The findings revealed a significant decrease in subchondral bone density at the location of the OCD/ OC lesion, particularly at the medial trochlear ridge. This area of low density was surrounded by a higher density rim. Furthermore, the contralateral joint showed a significantly higher overall mineral density.

**Conclusion:**

These results highlight the significant changes in bone mineral density associated with tarsocrural OCD/ OC. The lower density in the affected joint suggests pathological alterations in the subchondral bone, which may impact the bone turnover and contribute to the development of secondary osteoarthrosis, subsequently. The higher density observed in the contralateral joint emphasizes the role of altered joint loading and adaptation in the subchondral bone.

## Background

Osteochondritis dissecans (OCD) or osteochondrosis (OC) is known as a multifactorial developmental joint disease which may occur in different anatomical locations such as tarsus of the immature, rapidly growing large and giant breed dogs. However, a disruption in the endochondral ossification due to a failure of matrix calcification and vascular invasion [1] is considered to be the main pathogenesis of canine OCD/ OC [1–3]; a mismatch in biomechanical properties and change in a joint loading can be the start of a cascade leading to the development of these lesions [4–7].

Though the primary pathogenesis starts from cartilage which is radiolucent, the extension of the lesion and repeated focal stress which leads to significant changes in the integrity and density of the underlying subchondral bone. These changes of the subchondral bone can be detected by radiography or computed tomography (CT) [8, 9]. The underlying subchondral bone of a joint is characterized by its mineral density, collagen content, and ash fraction, which determine its biomechanical properties [10]. These properties can change due to modeling and remodeling of the bones that make up the joint, which are guided by local strains resulting from the joint’s biomechanical loading conditions. This ability to adapt is known as ”bone functional adaptation” [11, 12]. Among these properties, bone mineral density is an essential aspect of subchondral bone biomechanics that can be evaluated by CT. Despite the role of the bone density in presenting the biomechanics changes, there is limited knowledge available about the normal and abnormal subchondral bone density of talus in dogs [13, 14]. Recently published research has focused on reporting the normal and age-related subchondral bone changes in the talus of healthy Labrador Retrievers [15]. Previous studies have described the normal, age-related and degenerative changes in subchondral bone density distribution in the elbow joint [13, 16, 17], this information is lacking in the evaluation of the talus. Additionally, as the previous studies [17, 18] presented the increased subchondral bone mineral density measured in CT may be both a cause and an effect of OCD/ OC, the present study is designed to describe the subchondral bone density changes in Labrador Retrievers, as the predisposed breed to talar OCD/ OC [15, 19]. We hypothesized that the that the subchondral bone density changes will be significantly different between the affected limbs and unaffected limbs. The specific aims of this study are:

1. To evaluate changes in subchondral bone density in Labrador Retrievers with tarsocrural osteochondrosis using CT imaging.
2. To describe potential differences in bone density between affected and unaffected limbs within the same dog.

## Results

A total of eight dogs underwent a CT examination after clinical and radiographic diagnoses of unilateral OCD/ OC of tarsus. The age at the time of diagnosis ranged from 8 to 21 months (mean 13 months). Among the dogs, there were 5 males and 3 females. Two dogs had the advantage of having a follow-up CT available at 11 and 14 months after undergoing arthroscopic treatment for the OC lesion. These additional data were included in the study, as they presented an opportunity to assess changes in density distribution following arthroscopic treatment of tarsocrural OC lesions.

**Fig. 1:**
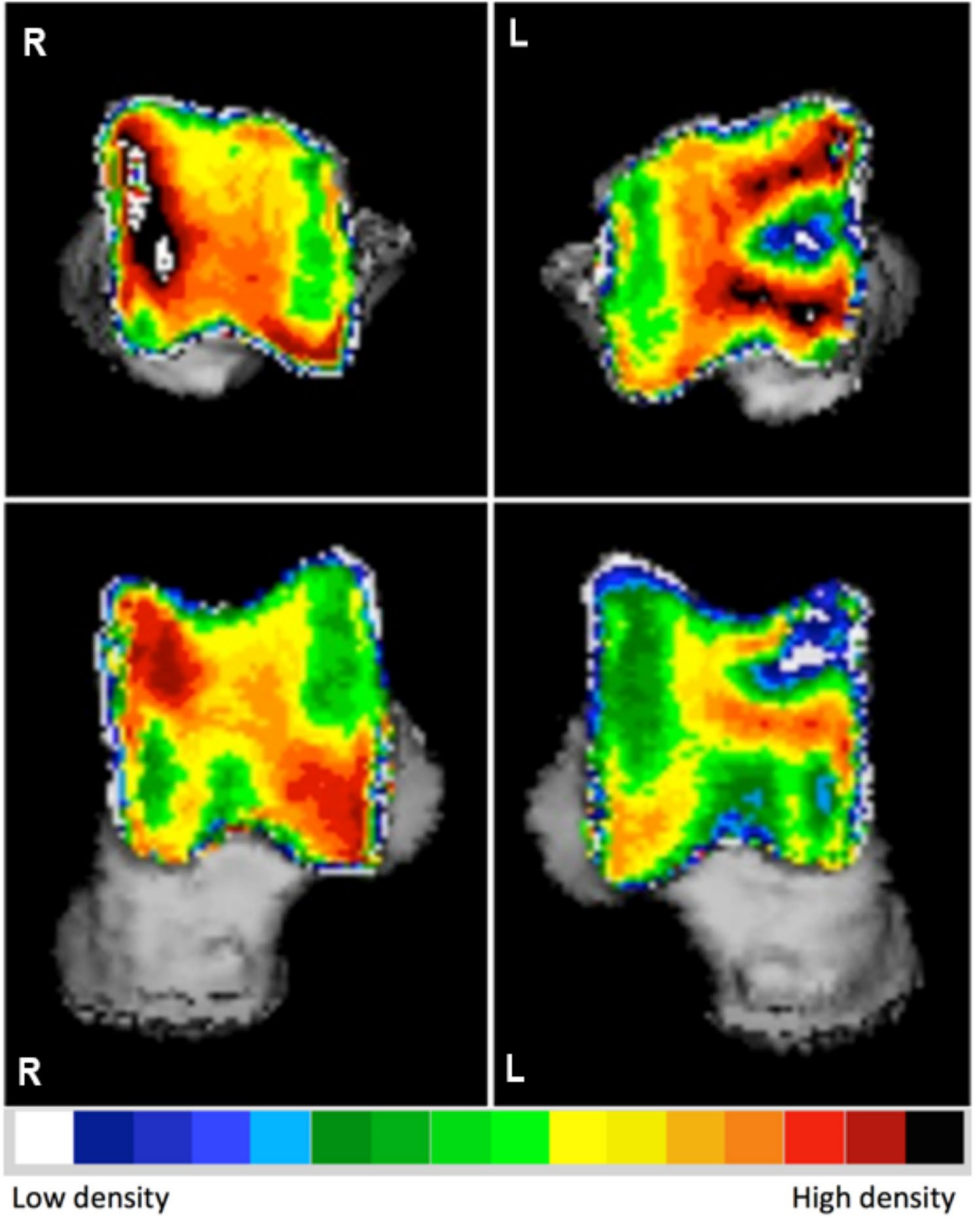
Densitograms of the right and left talus of a Labrador Retriever with medial trochlear ridge tarsocrural osteochondrosis on the left side. Note the appearance of the osteochondrosis lesion and the overall difference in density between the affected and the contralateral joint. L: left, R: right

The mean density of the lesion was reported as 612.6 ± 88.4 mg HA/cm ^3^ (mean ± SD) in all affected joints, a noticeable pattern emerged where there was a lower density observed at the specific location of the OC lesion, specifically at the level of the medial trochlear ridge. This area of reduced density was surrounded by a ring of increased density (Fig. 1). The proximal view provided the clearest visualization of the lesion due to its location on the proximal aspect of the medial trochlear ridge.

The density of the trochlear ridge was measured 780.7 ± 39.8 mg HA/cm ^3^ (mean ± SD) in the contralateral normal joint. When comparing the mean subchondral bone density between the affected joint and the contralateral normal joint, a statistically significant difference was observed (*P*-value of 0.001). The affected joint exhibited a significantly lower mean subchondral bone density compared to its contralateral apparently normal counterpart without clinical and radiographic signs of OCD/ OC.

Additionally, analysis of the subchondral bone density distribution in the contra-lateral normal joint showed the presence of two density peaks located at the proximal medial trochlear ridge and the distal lateral trochlear ridge in both proximal and dorsal views.

**Fig. 2:**
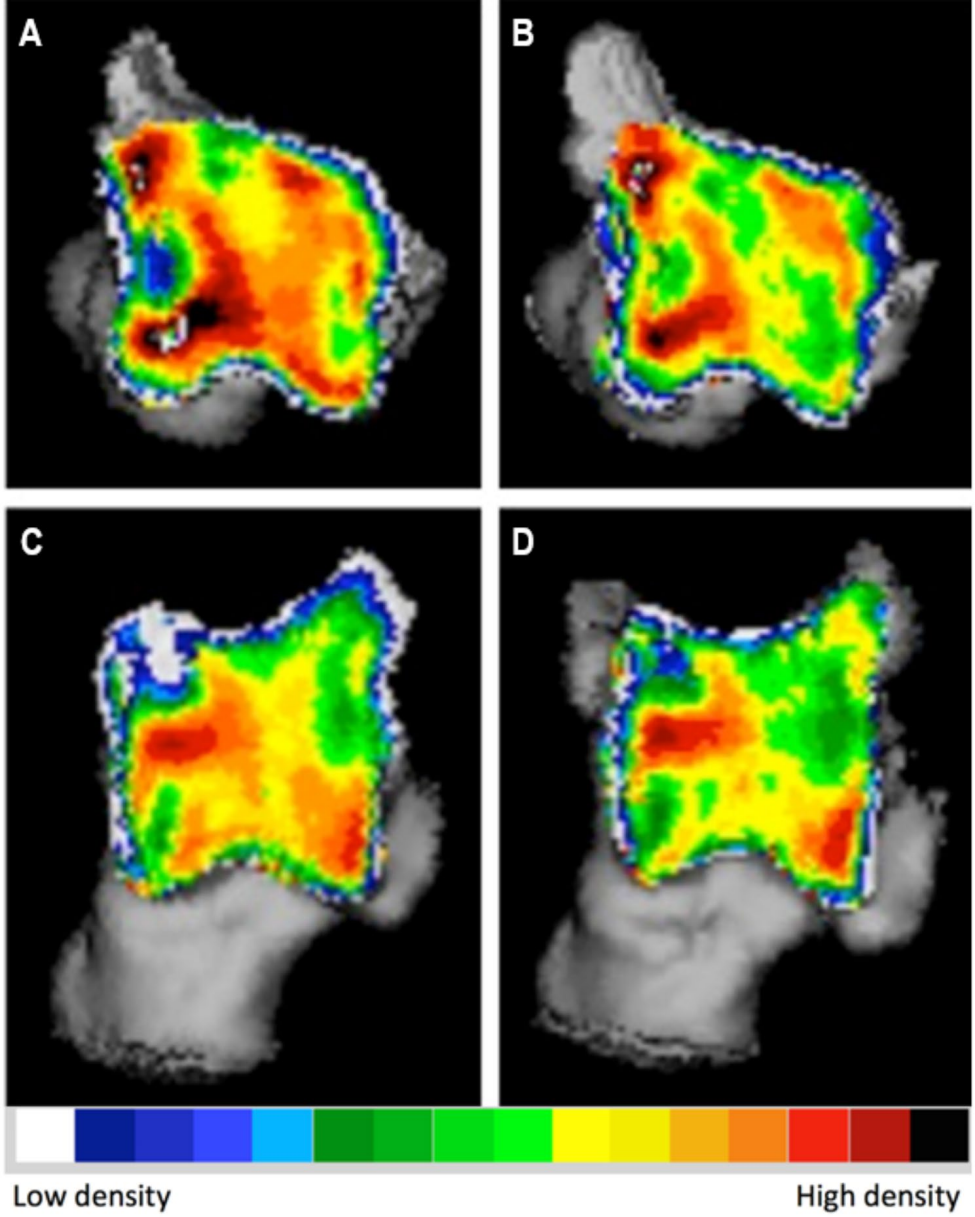
Densitogram of the talus of a Labrador with tarsocrural osteochondrosis of the medial trochlear ridge (MTRT-OC). On the left the subchondral bone density distribution at the time of diagnosis (**A** and **C**), on the right 14 months post-operatively (**B** and **D**). **A** and **B**: 3D reconstruction of the proximal view. **C** and **D**: 3D reconstruction of the dorsal view

**Fig. 3:**
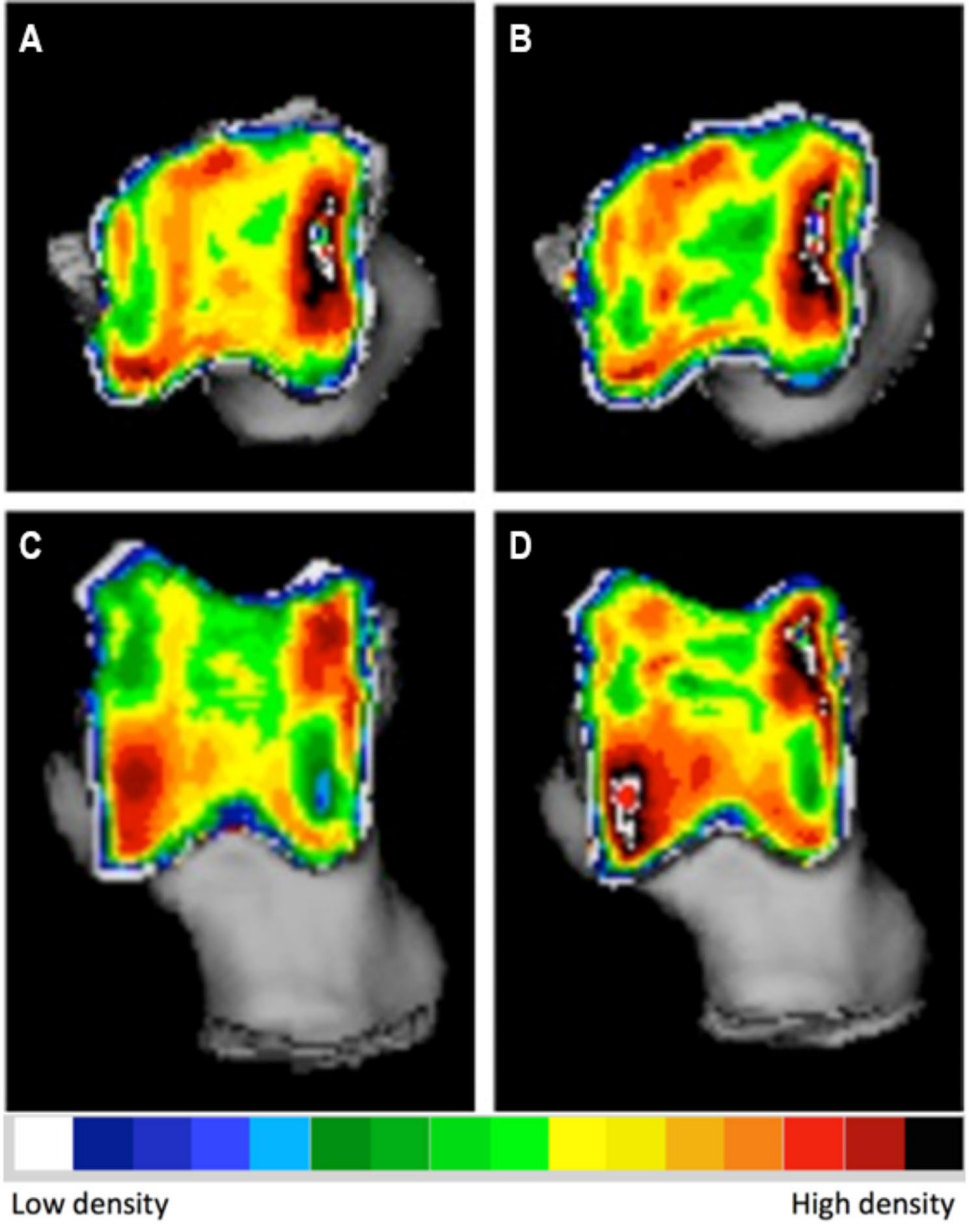
Densitogram of the contralateral of the talus of a Labrador with tarsocrural osteochondrosis of the medial trochlear ridge (MTRT-OC). On the left the subchondral bone density distribution at the time of diagnosis (**A** and **C**), on the right 14 months post-operatively (**B** and **D**). **A** and **B**: 3D reconstruction of the proximal view. **C** and **D**: 3D reconstruction of the dorsal view

The evaluation of subchondral bone density distribution overtime revealed notable differences between the two time points. Specifically, at the location of the subchondral bone defect, there was a reduction in the difference between the low-density defect and the high-density area surrounding it. In one of the dogs, there was a decrease in the mean subchondral bone density observed in the affected joint (Fig. 2). Conversely, the other dog exhibited an increase in mean subchondral bone density. In both dogs, the contralateral normal limb displayed an increase in both mean and maximum density (Fig. 3). These findings highlight the dynamic nature of subchondral bone density changes following arthroscopic treatment of tarsocrural OC lesions.

## Discussion

The present study revealed a significant decrease of the subchondral bone density at the location of the OCD/ OC particularly at the medial trochlear ridge which was surrounded by a higher density rim. Additionally, theoverall mean subchondral bone densityin the affected joint was lower compared to the contralateral normal side. These findings support our hypothesis, as we observed distinct alterations in bone mineral density associated with tarsocrural OCD. Therefore, and the study confirmed that subchondral bone density changes can occur overtime in affected joints, potentially influencing disease progression and joint adaptation.

A consistent observation across all affected joints in this study was the presence of a region with low apparent density, surrounded by a high-density area. This finding aligns with the characteristic subchondral bone defect associated with osteochondrosis with a sclerotic rim around the lesion, which can even be visible on radiographs in severe cases. These findings are in line with previous studies [2, 22] describing the CT characteristics of the OCD/ OC and the importance of the densitometry in evaluation of the intraarticular changes.

Additionally, previous in vitro experiments on bovine knees have demonstrated that as the size of a subchondral bone defect increases, the contact area around the defect adopts a rim-shaped pattern and expands. This finding aligns with our results, where we observed a higher-density rim surrounding the OCD/OC lesion. The formation of this rim-shaped contact area may reflect an adaptive response to altered load distribution, further supporting the relationship between mechanical stress and subchondral bone density changes in affected joints [23].

It is intriguing to note that changes in subchondral bone density distribution are not confined to the affected joint alone. In the contralateral tarsocrural joint, which was assessed as normal, both the maximum density and mean density were significantly higher than those observed in the affected joint. Tarsocrural osteochondrosis leads to lameness in the hind limbs, thereby altering joint kinematics and loading patterns. These altered loading conditions result in adaptive changes in the subchondral bone through modeling and remodeling processes [24, 25]. Specifically, lameness caused by tarsocrural osteochondrosis is characterized by a shorter stance phase in the affected limb, leading to a longer stance phase and increased loading of the contralateral leg.

These changes in loading can result in increased bone mineral density [26, 27] and the development of sclerotic areas as the bone adapts to the increased stress. Over time, these sclerotic areas may contribute to the development of secondary osteoarthritis due to altered biomechanics and increased joint stress. Therefore, understanding these pathophysiological changes in bone mineral density is crucial, as it may provide insights into the progression of osteochondrosis and the subsequent development of osteoarthritis. Based on Wolff’s law, bone adapts to the loads placed upon it by altering its density and structure [28]. Therefore, a reduction in bone mineral density and attenuation may indicate changes in the load distribution within the osseous structures of the subchondral bone under normal physiological conditions [28]. In other words, alterations in bone mineral density, which result in altered attenuation, can be seen as adaptive responses of the bone to changes in mechanical stress or load [25, 29]. This alteration of the density may present as a decrease or increase in the attenuation in computed tomography. A computed tomographic study quantifying the attenuation of the medial coronoid process suggested overall lower bone density [16, 30]. Humphreys et al. (2022) showed a significantly lower attenuation in the medial coronoid process in dogs with medial coronoid disease (MCD) compared with dogs without evidence of MCD; however, this finding had no relationship with the severity of the clinical signs [31].

In the context of compensatory contralateral joint loading, the changes observed in subchondral bone density align with Wolff’s law. When one limb is affected by tarsocrural osteochondrosis, leading to lameness, the contralateral limb undergoes increased mechanical stress due to compensatory weight-bearing. This increased stress triggers adaptive responses in the subchondral bone, leading to higher density and potentially sclerotic areas in the contralateral joint [25]. This phenomenon is supported by studies demonstrating that increased joint loading results in enhanced subchondral bone density [25]. The increased loading on the contralateral limb leads to an increase in subchondral bone mineral density as an adaptive response to the altered biomechanical environment, reinforcing the hypothesis of compensatory contralateral joint loading.

Furthermore, evidence from studies on other joints, such as the medial coronoid process, indicates that altered mechanical loading due to disease conditions can lead to significant changes in subchondral bone density and attenuation [13, 14, 16]. While these studies primarily focus on the affected joint, they underscore the broader principle that bones remodel in response to mechanical stress, supporting the notion that similar adaptive processes occur in the contralateral joints of dogs with tarsocrural osteochondrosis [16]. This aligns with our observation of higher subchondral bone density in the contralateral tarsocrural joint, emphasizing the role of compensatory mechanisms in response to altered gait and loading patterns.

While these kinetics of lameness are commonly understood, the data presented in this study demonstrate the morphological consequences at the level of the subchondral bone plate. This highlights one of the significant advantages of using CTOAM in the field of canine orthopaedic research: its ability to reveal and track morphological changes over time, enabling longitudinal evaluation of subchondral bone density.

In the present study, the subchondral bone density was obtained using CTOAM, a tool that enables the assessment of joint response to load and identification of regional changes. While global decreases in overall subchondral bone density would be unexpected in clinically healthy animals, it is important to note that CTOAM patterns can vary when a joint with altered contact mechanics responds to a standardized load [20]. However, changes in the joint biomechanics and repetitive microtrauma are considered as the causes of the OCD/ OC [2]. Changes of the normal biomechanics of the joint as a consequence of the OCD/ OC can accelerate the degenerative changes [18] and result in the subchondral density changes [21]. The main limitation of the present study is its retrospective nature. Therefore, while this study provides valuable insights, it is essential to consider its retrospective design when interpreting the findings and drawing conclusions. Further research using prospective study designs and rigorous methodologies would be beneficial to confirm and expand upon these findings.

Another notable limitation of this study is the small sample size, which was inherently constrained by the retrospective nature of the study and the reliance on referral cases. As a retrospective study, we were limited to the available clinical records and imaging data of Labrador Retrievers diagnosed with tarsocrural osteochondrosis at our referral centre. This restriction resulted in a smaller cohort than what might be ideal for broader generalization of the findings.

The small sample size may impact the statistical power of the study, potentially limiting the ability to detect subtle differences or correlations. Although we conducted a power analysis to ensure that the study could detect significant effects within the available sample, the results should be interpreted with caution. The findings provide valuable preliminary insights but necessitate validation through larger, prospective studies to confirm the observed trends and to enhance the robustness of the conclusions.

Additionally, the use of referral cases might introduce a selection bias, as these cases often represent more severe or atypical presentations of the condition, potentially skewing the results. This limitation underscores the need for further research involving a larger and more diverse population to fully understand the subchondral bone density changes in Labrador Retrievers with OCD/ OC and to verify the compensatory mechanisms in contralateral joints.

Based on the previously reported studies in the human and veterinary medicine [32, 33], an increase in bone density would be expected to enhance its compressive strength which may explain the increased density of the subchondral bone in the contralateral normal joint as a response to increased compensatory loading conditions.

As previously reported in animals affected by medial coronoid disease, a reduction in bone density can be a result of abnormal remodeling of the subchondral bone [34]. This condition disrupts the normal process of endochondral ossification in the calcifying zone of the articular cartilage, leading to weakened areas between the hyaline cartilage and subchondral bone [35]. The repetitive strain caused by weight-related factors contributes to the formation of microfractures in the bone [36]. In healthy conditions, these microfractures would trigger physiological bone remodeling, with osteoclasts removing damaged tissue and osteocytes promoting new bone formation [36–38].

The present study has some limitations that should be considered. Firstly, the retrospective design of the study limits the control over data collection and introduces potential biases. Furthermore, the absence of a control group of normal dogs prevents direct comparisons of subchondral bone density between affected and unaffected joints. Additionally, because the follow-up control was conducted only in two dogs, the results should not be regarded as a definitive outcome. Instead, this topic should be noted as a subject for further evaluations and research designs. The study also lacks an assessment of the correlation between density changes and the severity of clinical signs, which could provide valuable insights into the clinical relevance of the observed bone density alterations. However, the radiographic examination of the joint is normally performed in clinically affected dogs. It remains uncertain whether OCD/ OC can exist in a sound state without clinical manifestations and, subsequently, without obvious radiographic and CT changes. Therefore, the results presented in the current research should only be extended to clinically affected dogs. These limitations highlight the need for larger prospective studies with control groups and comprehensive clinical evaluations to further investigate the subchondral bone density changes in tarsocrural OCD/ OC and their clinical implications.

## Conclusion

In conclusion, a significant change in the subchondral bone mineral density should be expected in the canine tarsocurral joint affected with OCD/ OC as a cause or result of the lesion. Additionally, significant higher mineral density should be expected in the contralateral joint possibly as a consequence of change in the load balance and weight-bearing.

## Materials and methods

### Study population

An initial population of Labrador Retriever assistance dogs, which were presented at the Faculty of Veterinary Medicine, Ghent University, Ghent, Belgium from 2014 to 2017 for computer tomographic examination of the elbow and tarsal joints for screening of elbow dysplasia and tarsocrural osteochondrosis was included in this study. The study was approved by the ethical committee of the Faculty of Veterinary Medicine, Ghent University (approval nr. EC2011/193 and EC2014/186). The dogs were supplied by an assistance dog training organisation. A written owner consent was obtained in each case.

**Fig. 4:**
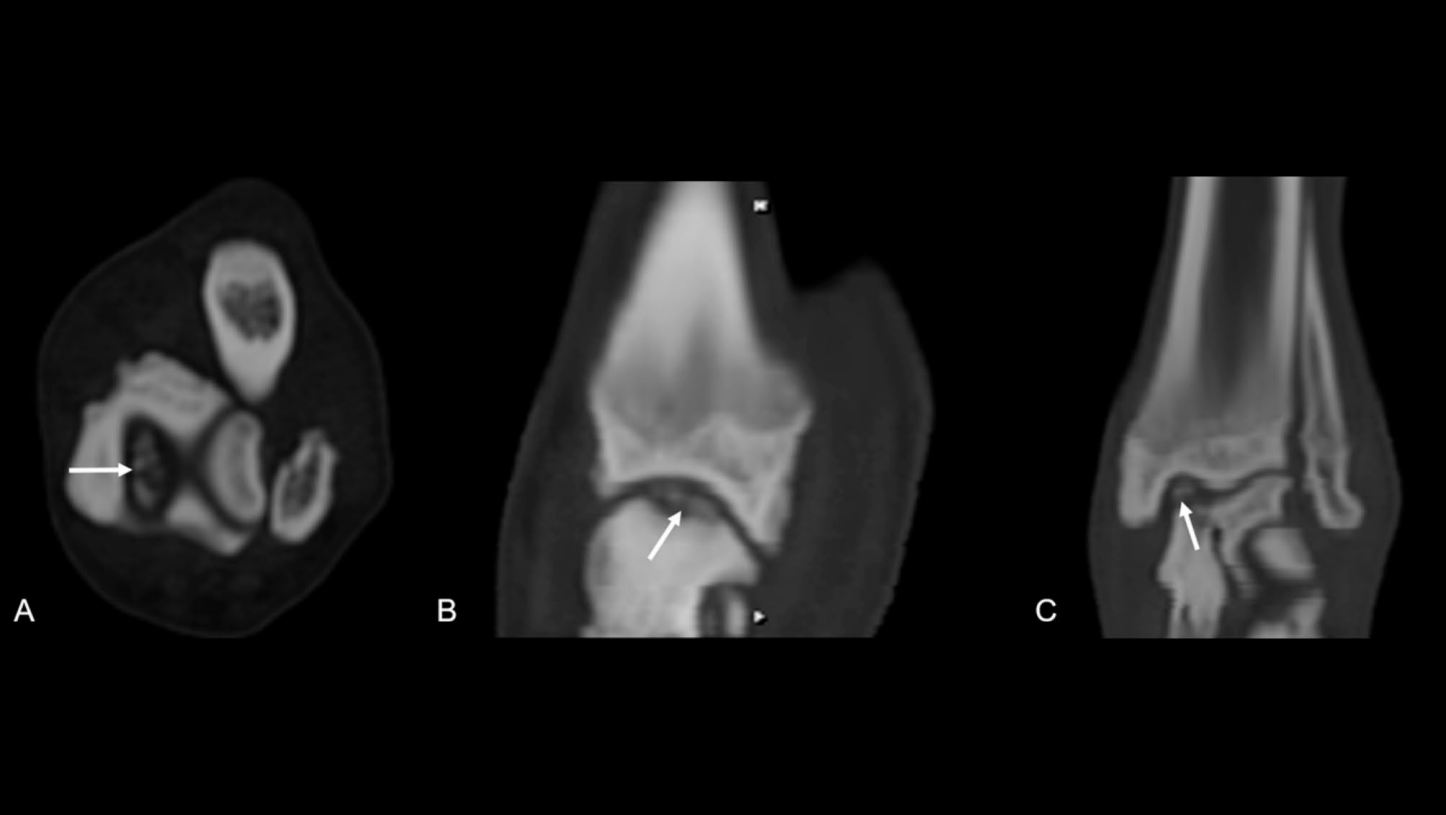
Transverse (**A**), sagittal (**B**) and dorsal (**C**) reconstructed image of a medial tarsocrural osteochondrosis lesion (white arrows). Multiple detached fragments can be seen

The study had a retrospective design which dogs diagnosed with unilateral tarsocrural osteochondrosis of the medial trochlear ridge (MTRT-OC) were included (Fig. 4). The inclusion criteria required complete orthopedic and CT examination, while dogs with only one tarsus examined by CT were excluded. If follow-up CT examinations were available for any of the cases, they would be included in the evaluation to assess changes over time.

### Image acquisition

All CT examinations were done under general anesthesia, using an IV bolus of 2 mg/kgBW propofol (Propovet, Schering-Plough, Comazzo, Italy) and intubated. Anesthesia was maintained with isoflurane (IsoFlo, Abbott Laboratories, Queensborough, UK) in 100% oxygen. CT scans were performed by a four-slice scanner (Lightspeed Qx/I, General Electric Medical Systems, Milwaukee, WI) using 120 kVp, 300 mA, 1.2 mm thickness collimated with overlap of 50% from distal part of the tibia/fibula to the toes. The images were reconstructed with a bone and a soft tissue reconstruction algorithm.

The scans were performed with the dogs positioned in ventral recumbency and left and right tarsal joints were scanned simultaneously, according to patient protocol and previous publications with the tarsal joints in extension [15, 21]. The optimal positioning was confirmed on the laterolateral and dorsoplantar scout view. The entire acquisition process, including positioning, took approximately five minutes. A calibration phantom (Bone Density Calibration Phantom, QRM GmbH, Germany) was placed between the scanning table and the tarsal joints as a density reference standard.

### Image analysis

**Fig. 5:**
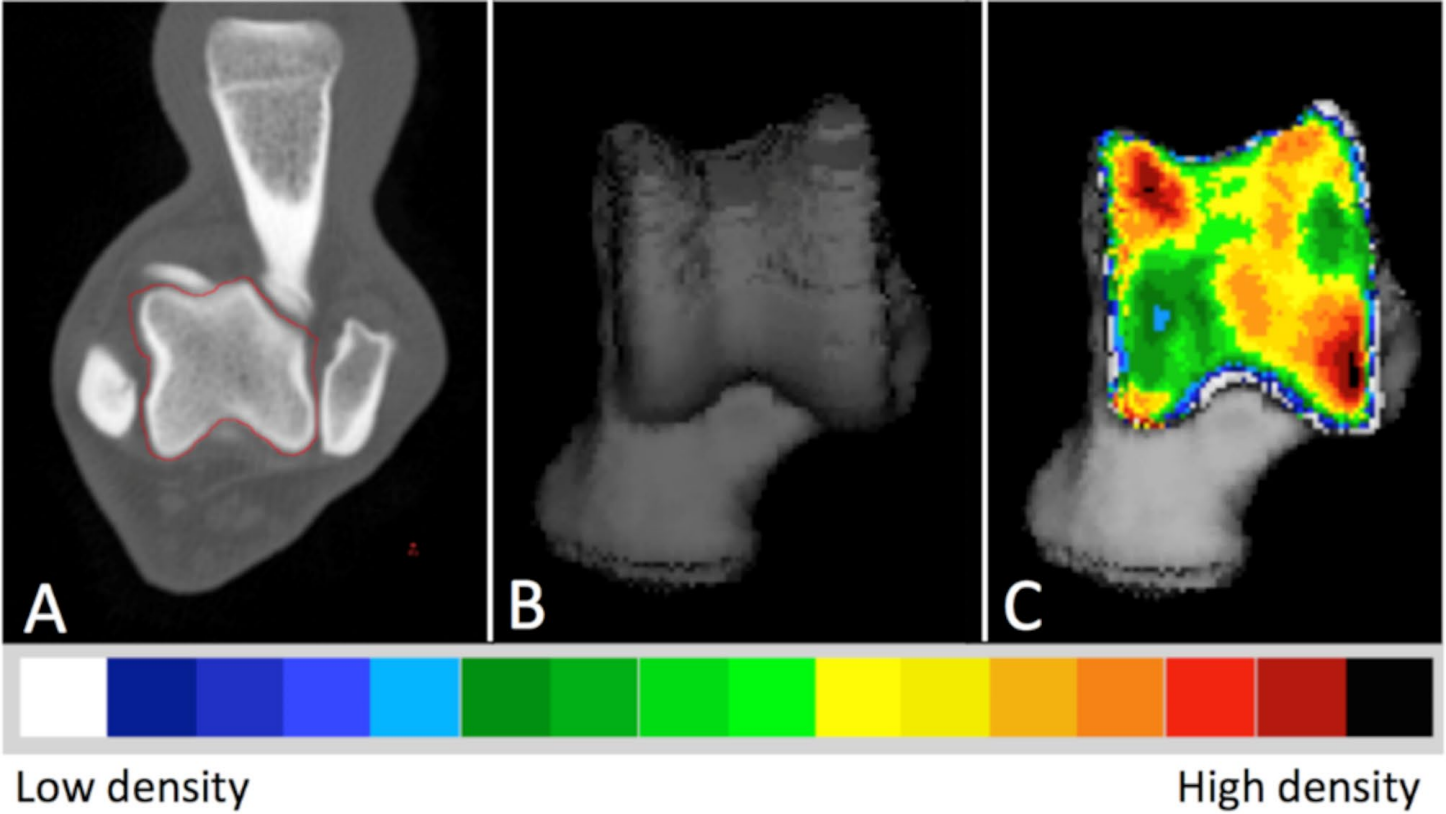
The Computed Tomography osteoabsorptiometry (CTOAM) workflow. Segmentation (isolation) of the talus bone on transverse images (**A**), 3D reconstruction of the dorsal view (**B**), and the MIP with false-colour scale visualizing the distribution of the subchondral bone density on a dorsal view of the talus (**C**)

**Fig. 6:**
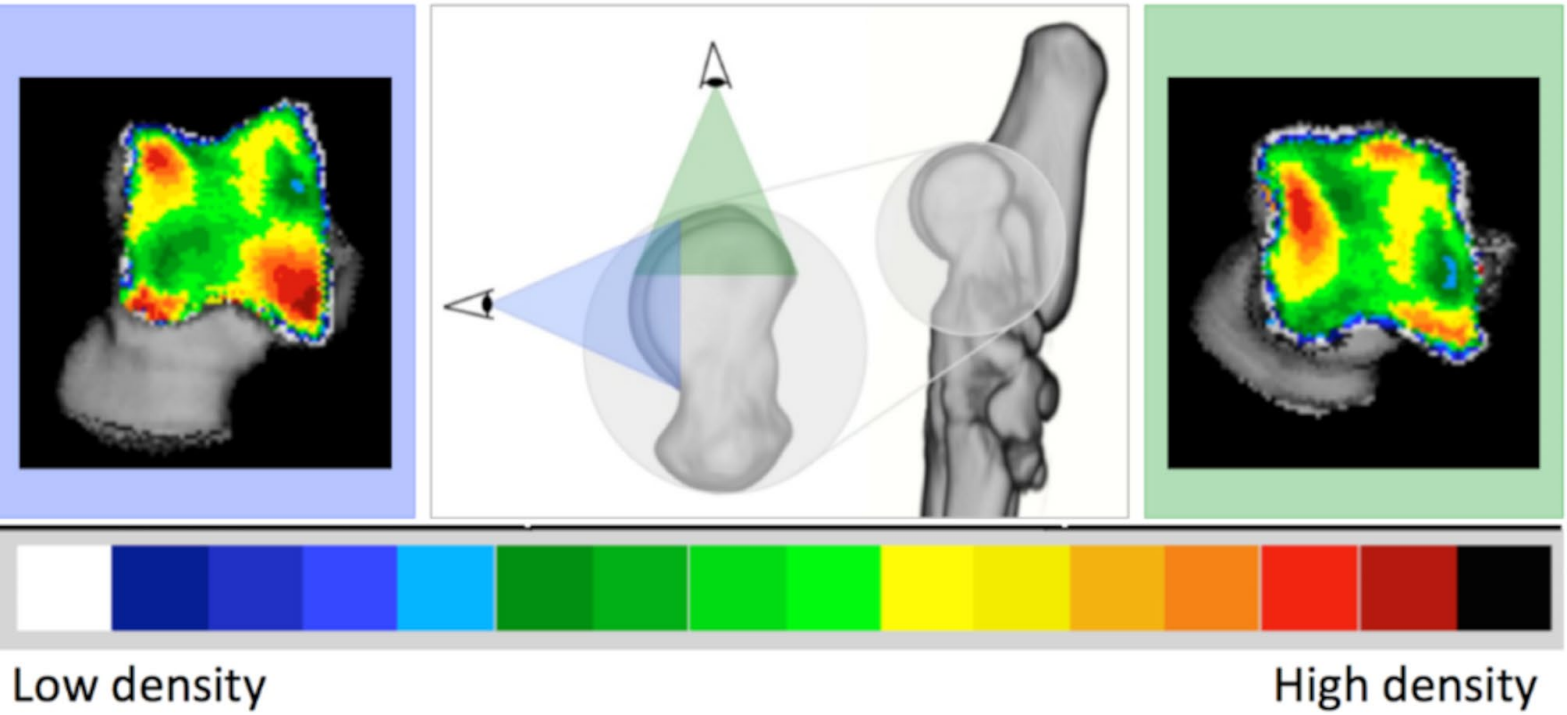
Three-dimensional reconstruction of the tarsal and metatarsal bones of the right foot, medial view. Line of sight for the two 3D reconstructions that are reconstructed from the segmented images, proximal view (green) and dorsal view (blue). The use of these two views provides full visualisation of the trochlear ridges with the typical distribution shown for the proximal (right) and dorsal (left) view

CT images of the included cases were exported in DICOM format to the Analyze 11.0 software (Biomedical Imaging Resource, Mayo Foundation, Rochester, MN, USA) to complete the CTOAM workflow, as illustrated in Fig. 5 and is previously reported by the same authors [15]. Initially, the talus was segmented using the software’s segmentation algorithm. Two different three-dimensional (3D) views of the trochlear ridges were then reconstructed based on these segmented images (Fig. 6). The proximal view was reconstructed first, and the dorsal view was obtained by rotating the proximal view backward by approximately 90 degrees. This method enabled a thorough evaluation of the entire proximal talar subchondral bone, including both the lateral and medial trochlear ridges (Fig. 6). Subsequently, the subchondral bone plate of the articulating surface was isolated and reconstructed in the same orientations.

The maximum bone density was projected onto the articular surface using a maximum intensity projection (MIP). In this step, the 3D data volume (in voxels) of the subchondral bone plate is converted into a 2D image (in pixels), with each pixel representing the maximum value based on Hounsfield Units (HU). This value is obtained from the voxels along a line perpendicular to each pixel in the 2D image. The length of this line, or the MIP depth, was determined by the thickness of the subchondral bone plate and was set to 1.5 mm. The mean density was calculated accordingly from the HU of the included pixels. The MIP view was then transformed into a false color scale, where the HU range of 200 to 1200 was divided into 100 HU intervals, each represented by a different color. The colors, in descending order, were black, dark red, light red, orange, yellow, dark green, light green, dark blue, light blue, and white. This process resulted in a densitogram (Figs. 5 and 6), which displays lines of equal density (isodensities). The densitogram visually represents the apparent density distribution and was further analyzed.

For quantification purposes, the density values (in HU) were converted to 8-bit values, creating 256 density values, which were then evenly distributed into eight bins according to the literature [36]. Each bin contained 32 density values. A density maximum was defined as an area with density values in the two highest density bins of the densitogram. To compare individual subchondral bone density distributions, a 30 × 30 unit grid was superimposed on the densitogram of both the proximal and dorsal views of the trochlear ridges. The grid edges were positioned to ensure the entire joint surface fit within. The number of units in each grid remained constant to standardize the coordinates of the density maxima. The x- and y-coordinates were used to describe the location of the density maxima on the joint surface.

Furthermore, the size of the density maximum was expressed as a ratio of the area of the density maximum to the joint surface area in both the proximal and dorsal views, defined as the maximum area ratio (MAR). The use of MAR allows a relative comparison between individuals, and accounts for size-differences. MAR was calculated as follows:

MAR = number of pixels of the density maximum ÷ pixels of the total joint surface

Considering the potential influence of joint loading on the subchondral bone density distribution, it was acknowledged that the contralateral joint might also experience changes despite the presence of a unilateral osteochondral (OC) lesion. Therefore, densitograms were created for both the affected joint and the contralateral joint in order to capture the density distribution in both cases.

For dogs that underwent follow-up CT studies, the mean density and maximum density were compared between the affected joint and the contralateral joint at both time points.

### Statistics

Statistical analysis was performed using SPSS (version 19.0; IBM, Chicago, USA). The mean and maximum density values obtained from both the affected and contralateral joints were compared. To ensure the statistical validity of the analysis, the data was first assessed for normality using the Shapiro-Wilk test. Subsequently, a paired Student’s t-test was performed to further evaluate the data. A significance level of *P* < 0.05 was set to determine statistically significant differences between the two sets of measurements.

## Data Availability

The data are available and will be provided by the corresponding author with a reasonable request

## Abbreviations

CTOAM: Computed Tomography osteoabsorptiometry
MCD: Medial coronoid disease
MTRT-OC: Tarsocrural osteochondrosis of the medial trochlear ridge
OC: Osteochondrosis
OCD: Osteochondritis dissecans
